# Using the Inverse Three-Point Bending Test to Determine Mechanical Properties of Plant Stems

**DOI:** 10.3390/mps8020032

**Published:** 2025-03-18

**Authors:** Alexander Anisimov, Maksim Suslov, Anna Petrova, Tatyana Chernova, Oleg Gorshkov, Tatyana Gorshkova

**Affiliations:** Kazan Institute of Biochemistry and Biophysics, FRC Kazan Scientific Center, Russian Academy of Sciences, P.O. Box 30, Kazan 420111, Russia; anisimov@kibb.knc.ru (A.A.); anna.petrova@kibb.knc.ru (A.P.); chernova.t@mail.ru (T.C.); o_gorshkov@mail.ru (O.G.); gorshkova@kibb.knc.ru (T.G.)

**Keywords:** macromechanical properties, inverse three-point bending test, plant stems, flax, elasticity modulus

## Abstract

Biomechanical parameters of plant tissues and organs are increasingly recognized as key factors in plant development and application, increasing the demand for convenient devices for their study. The paper presents an original device for performing a three-point bending test using the inverse method, which is a modification of the classical (straight) three-point test. The designed device was tested in experiments to determine the modulus of elasticity of flax plant stems, and the results were compared with data obtained using the vibration method and the straight three-point bending test on a commercial instrument. Due to the high sensitivity associated with its design features, the device for the inverse three-point bending test is characterized by being able to adequately measure elastic moduli in plant stems over a wide range of values, from tens of MPa to tens of GPa. It also allows checking the effect of humidity, temperature, and water content on the mechanical properties of samples and is equipped with an automation system. The proposed device is quite affordable and can be effectively used both for young stem parts, whose mechanical properties are based on a hydroskeleton, and for mature, poorly hydrated parts with cell walls highly developed in sclerenchymatous tissues.

## 1. Introduction

Biomechanics is a rapidly growing field of science that studies fundamental aspects of the relationships between mechanical properties, architecture, and functional morphology of plants, including in response to external influences [1,2,3,4,5,6,7]. Plant breeders and molecular biologists are using biomechanical data more frequently to phenotype plants, especially transgenic or cell wall mutant plants [8,9,10]. The results of biomechanics research are in demand for the design of working elements of agricultural machinery that are optimal for efficient plant harvesting and processing [6]. Another practical application of plant biomechanics data is the use of plant fibers to reinforce polymers [11,12,13]. Estimating the elastic properties of plant organs and tissues that can vary in a broad range is a common challenge in plant biomechanics.

Methods for assessing the mechanical properties of biological tissues are similar to those used for engineering structural materials. Considerable attention in the plant biomechanics literature has typically focused on methods for estimating the elastic modulus associated with the application of an external force (stress) along or across the specimen. Common methods include bending and axial loading tests, such as tension and compression tests [14,15]. Various methods are used, including the two-point (cantilever) [5], three-point [16,17], and four-point [18] methods, as well as methods that use compression and tension of the specimen [19]. The latter are widely used in materials science. However, when applied to plant samples, including plant stems, their disadvantage is the need for rigid fixation of the sample. Almost all of the above methods can be used in cyclic loading (fatigue) tests. Fatigue tests are performed by repeatedly applying alternating(cyclic) loads to a specimen. Such tests determine the number of cycles until failure of specimens at different stress levels [20,21,22]. Mechanical properties can be investigated using vibrations since each object has its own vibration frequency, which depends on its stiffness and mass [10,23]. However, this method has some limitations; for example, the specimen under investigation must have a rather narrow stiffness level, below and above which it is very difficult to estimate the vibrations.

**Figure 1:**
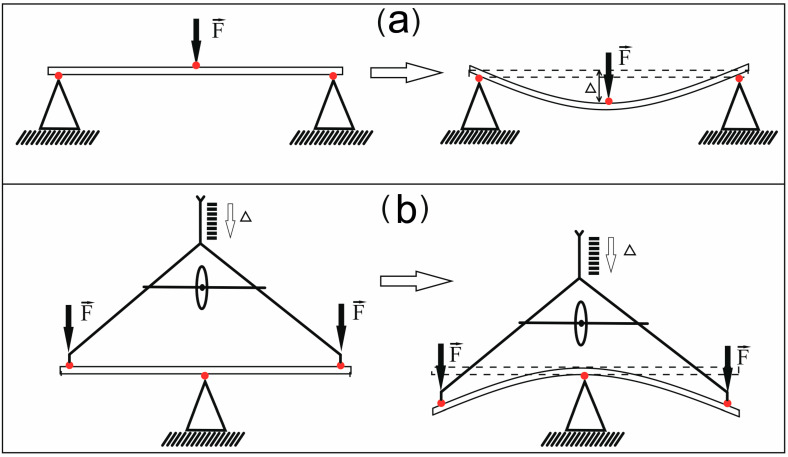
Schematic variants of the three-point bending test: (**a**) Classical three-point method; (**b**) inverse three-point method. F is the load; ∆ is the sample bending magnitude. The red dots in the figures indicate the points of force application.

The classical three-point bending test is widely used to determine the elastic modulus of plant stems due to its simplicity, clarity, and the fact that it does not require rigid fixation of the specimen [16,17,18,24,25,26]. The method involves recording the bending magnitude of the stem ∆, whose ends are placed on two fixed (base) support prisms, as a function of the magnitude of the load F applied to the center of the specimen (Figure 1a). However, in the three-point bending test, the largest bending deflection is at mid-length, and the highest shear is near the supports. Shear effects have influence in bending tests when the length-to-diameter ratio (where length is the distance between supports, and diameter is representative of a diameter or thickness of the stem) is not large. Shear leads to an underestimation of elastic modulus [15]. This is especially important for anisotropic materials when testing in the fiber direction. Ideally, the length-to-diameter ratio should be greater than 20 to avoid underestimating the mechanical properties [15]. Currently, commercial three-point bending tools are either very large scale, with study areas in the range of GPa, or very expensive, with sensitive strain gauges.

In the present study, we attempted to develop an inexpensive device for the three-point bending test without loss of accuracy and sensitivity of measurement and proposed a modification of the classical three-point method (hereinafter referred to as the inverse three-point bending test). The essence of the modification lies in the rearrangement of the argument and the function. In the classical method, the load is first applied to the center of the stem, the ends of which are placed on two fixed supporting prisms, and the amount of bending is then measured (Figure 1a). In the inverted version, an accurately controlled displacement of the entire sample is first applied to a fixed support prism (Figure 1b), which is placed on sensitive scales that record the magnitude of the load. The inverted three-point bending test allows the bending value of a specimen to be accurately measured in small increments, starting at tens of microns, and the resulting stress to be measured using portable digital scales.

The constructed device was tested by the stepwise loading method on samples of developing flax stemswithdifferent moduli of elasticity and oat coleoptiles. The obtained results were compared with the data obtained using other approaches to determine the mechanical properties of specimens: the straight three-point bending test with constant load [27,28] and the vibration method [10,29].

## 2. Material and Methods

### 2.1. Plant Material

Flax plants (*Linumusitatissimum* L.) of long fiber varieties Mogilevsky and Bolshoy and oilseed variety Bethune were grown in pots with a 50-cm soil layer under natural light (55°47′33.7″ N 49°07′15.8″ E, Kazan, Russia) with daily watering. Forty-day-old (approximately 30 cm in height) plants, fifty-day-old (approximately 50 cm in height) plants, and mature plants (75 cm high) were used for measurements depending on the experiment. The upper part of the developing flax stem is easy to tear, while the lower part requires greater force to break [30,31]. The place of a sharp jump in the strength characteristics of stems is called the snap point [31]. The flax samples collected above and below the snap point were used to check the sensitivity of the constructed device. Seeds of oat (*Avena sativa*, cultivar Skakun) were sterilized using 0.35% NaOCl solution (10 min), washed three times with distilled water, and then grown hydroponically for 10 d. Coleoptiles from 10-day-old oat seedlings were used to evaluate mechanical properties.

### 2.2. Mechanical Properties Investigation

The studies were carried out using a designed inverse three-point test machine (the design of the machine is given in the Results and Discussion, Section 3.1), a MultiDrive MCR 702e rheometer (Anton Paar, Graz, Austria), and a vibration method [10] adapted for flax stems [29]. Segments of the coleoptiles or stems for macromechanicalpropertydetermination were cut immediately before the measurement to minimize the effect of water loss on the sample. The experiments were carried outfirst on the defoliated stem segment and then, if indicated, on the xylem part of the same segment obtained after peeling off the phloem part. Fresh weight (FW) values were measured for each sample, and anatomical analysis was performed on hand transverse sections taken right above and below the segment under study [29]. All samples were analyzed with a Leica DM1000 microscope (Leica Biosystems, Germany) and photographed with a digital camera.

#### 2.2.1. Inverse Three-Point Bending Test

The three-point bending test was carried out using an inverse three-point test machine (Section below “Design of the device for the inverse three-point bending test”). Four-cm flax (Bolshoy variety) stem segments, cut 13 cm above the cotyledons, were used in the experiments to establish the inverse three-point bending tests and compare its results with other methods evaluating the stem macromechanical properties. To compare the different parts of the developing flax stem (Mogilevsky variety), 4-cm-long stem segments, cut 1.5 cm below apex and 5 cm above the cotyledons, were used. To compare the properties of the same stem portion at different stages of plant maturation, 7-cm-long flax (Bethune variety) stem segments, cut 4 cm above the cotyledons from forty- and fifty-day-old plants, were used, as well as 7-cm segments from the middle stem part of mature plants (Mogilevsky variety). To evaluate the mechanical properties of monocotyledonous plant fragments, 1.5-cm-long oat coleoptile segments were used. The experiments were conducted at a constant temperature of 25 °C and 50% humidity. The increase in load was done in steps; step of displacement was 125 μm or 250 μm, and the break was 30 s. The total displacement was 1250–2500 μm, depending on the experiment.

The values of Young’s modulus *E* were determined using Equation (1):

(1) $$E=\frac{{FL}^{3}}{48\Delta LI},$$

where *F* is the load value, *L* is the stem length, ∆*L* is the bending value, and *I* is the axial moment of inertia of the stem.

For cylindrical specimens with internal cavity, *I* was obtained using Equation (2):

(2) $$I=\pi \frac{\left(D^{4}-d^{4}\right)}{64},$$

where *D* is the external diameter of the stem/xylem and *d* is the diameter of the internal cavity. External stem diameters ranged from 1.2 mm (areas above the snap point) to 2 mm (areas below the snap point). External diameters of xylem ranged from 1.2 to 2 mm. External diameters of coleoptile were about 1.3 mm.

#### 2.2.2. Three-Point Bending Test with Rheometer

A three-point bending test was carried out using aMultiDrive MCR 702e rheometer (Anton Paar, Austria) with the MHR-100 humidity control module and the CTD 180 convection temperature device. Three-cm-long flax (Bolshoy variety) stem segments, cut 13.5 cm above the cotyledons, were used for the three-point bending tests. The experiments were conducted at a constant temperature of 25 °C and 50% humidity. The loading rate of the sample was 5–50 µm/s for the areas above the snap point and 3.9 µm/s for the area below the snap point. The increase in load was done constantly. The values of Young’s modulus *E* were determined using Equation (1).

#### 2.2.3. Vibration Method of Mechanical Properties Investigation

Ten-cm-long flax (Bolshoy variety) stem segments, cut 10 cm above the cotyledons, were used for the vibration method. Macromechanical properties were investigated using a Python 3.12 script for vibration evaluation [10,29].

The following equations were used to calculate the mechanical properties.

The stiffness *k* of the sample was calculated using Equation (3):

(3) $$k=(2\pi \eta {)}^{2}m_{eq},$$

where *k* is the stiffness, *η* is the natural frequency, and *m_eq_* is the equivalent mass of the oscillator (Equation (4)):

(4) $$m_{eq}=\delta \times 0.9\times FW,$$

where *δ* is the coefficient depending on the taper ratio,0.9 is the coefficient, which means that only 9 out of 10 cm of the sample participated in the vibration, *FW* is the fresh weight of the sample, and *δ* = 0.245 in the case of a rod showing a uniform weight distribution [10].

The stiffness was converted to Young’s modulus Eusing Equation (5) [23]:

(5) $$k=\frac{nEI}{L^{3}}$$

where *k* is the stiffness, *E* is the Young’s modulus, *I* is the moment of inertia, *L* is the length of a specimen, and *n* is the coefficient, depending on the taper ratio. Equation (5) was used to calculate *E_stem_* and *E_xylem_*.

### 2.3. Statistics

To evaluate mechanical properties, 10 plants were used for each experiment. A total of 70 stem fragments, 50 xylem fragments, and 10 coleoptile fragments were examined. Means with standard deviations among biological replicates are presented. Separation of means was performed with one-way ANOVA followed by *t*-test or Tukey test at α = 0.05 using SPSS software package (v.21, IBM Corp, Armonk, NY, USA).

## 3. Results and Discussion

### 3.1. Design of the Device for the Inverse Three-Point Bending Test

**Figure 2:**
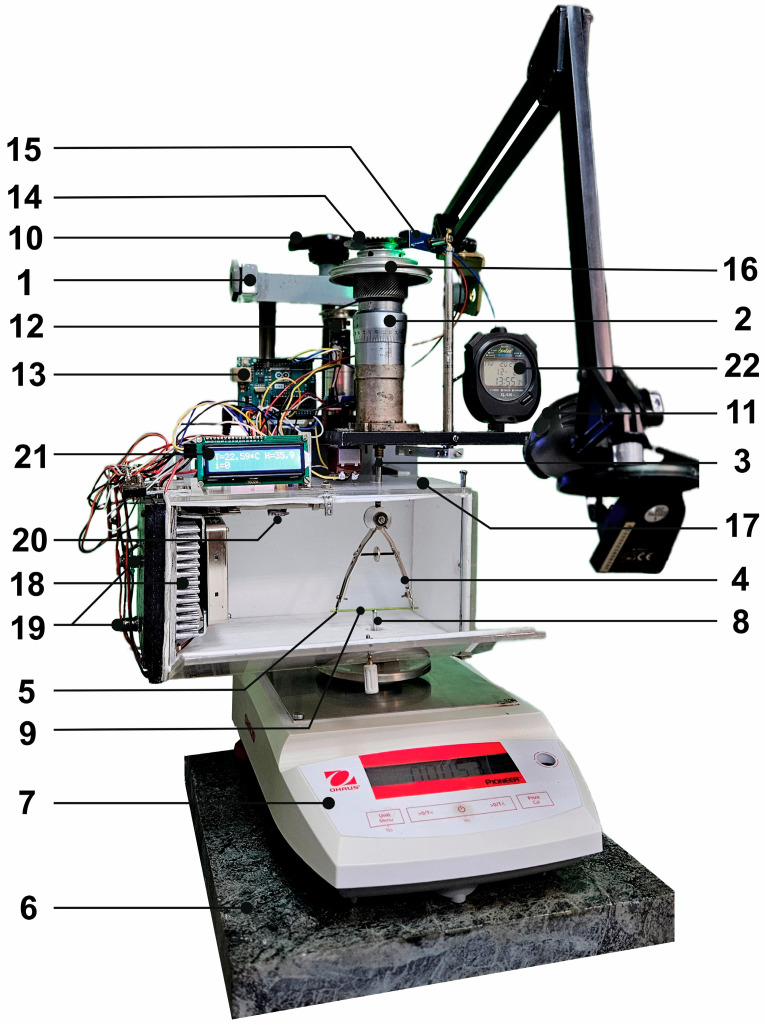
The design of the device for the inverse three-point bending test: 1—cremalier; 2—mechanical micrometer; 3—movable plunger; 4—V-shaped sample holder; 5—stem segment holding rings; 6—vibration-absorbing ceramic granite plate; 7—analytical scales; 8—support prism; 9—stem segment; 10—screw lift; 11—video camera; 12—DC motor with a gearbox; 13—ArduinoUno R3 hardware and software platform; 14—metal disc with slots; 15—optocoupler; 16—four-stage pulley; 17—thermostatically controlled chamber; 18—needle radiator; 19—water cooled radiator; 20—temperature and humidity sensor; 21—digital display; 22—stopwatch.

The design of the device for the inverse three-point bending test can be divided into two main parts—mobile and fixed (Figure 2, Supplementary S1). The mobile part is a mounting platform mounted on the screw lift of the cremalier (1), which moves along the vertical guides fixed to the horizontal base of the cremalier. A mechanical micrometer (2) is mounted rigidly on the mounting platform, the movable plunger (3) of which is extended by a rod with a longitudinal keyway. The rod is chiseled from the end beneath the size of the micrometer plunger and is sprung to the plunger jaw through a steel ball. The ball, acting as a bearing, is required to ensure tight contact in the longitudinal direction while maintaining free rotation of the plunger in relation to the rod. The rod is then connected to a V-shaped sample holder (4), which is fixed to the rod.

At the ends of the V-shaped holder, there are stem segment holding rings (5), into which a horizontally oriented sample (stem segment) is inserted. Turning the micrometer screw causes a forward vertical movement of the plunger, the V-shaped holder and, consequently, the specimen, allowing the amount of specimen deflection (Δ) to be adjusted during the three-point bending test.

The stationary part of the device consists of a vibration-absorbing ceramic granite plate (6) and OHAUS PA214C analytical scales (Ohaus Corporation, Parsippany, NJ, USA) (7) mounted on it. An aluminum support prism (8) is mounted on the weighing scales platform, on which the center of the stem segment stands during the experiment. In this way, the analytical scales mounted on a fixed platform performs the function of a load sensor. The scales are equipped with a digital display and a serial port for data transmission using the RS-232 protocol. The accuracy of the scales and the maximum load are 0.0001 g and 220 g, respectively. An important condition for the selection of the scales was that the scale plate should not deflect under load.

For the three-point bending test, a stem segment (9) of the required length is inserted into the rings of the V-shaped holder. Either before or after this, the angle of the V-holder solution is adjusted continuously, thereby setting the reference length of the specimen (L), i.e., the distance between the two reference points at the ends of the specimen. The length L is adjusted while maintaining the position of the center of the sample at the point of contact with the support prism. The screw lift (10) is used for rough and fast preliminary movement of the specimen towards the support prism. It is advisable to use a screw lift to avoid uncontrolled accidental displacement of the mounting platform with the micrometer. In turn, the micrometric feed is used for smooth contact of the specimen with the support prism, the moment of which is controlled by the scales. The micrometer is then used to adjust the bending value step by step, and the load value is recorded on the scales. A video camera (11) records the course of the experiment. This makes it possible, if necessary, to repeatedly observe changes in the state of the specimen during the three-point bending test, environmental parameters, etc.

In order to simplify the experiments and to eliminate errors and inaccuracies in the setting of the parameters related to the human factor, the device was equipped with an automation system. The automation system includes an active element in the form of a DC motor with a gearbox (12) under the control of electronics based on the ArduinoUno R3 hardware and software platform (“Arduino AG”, Turin, Italy) (13). The motor (12) is inserted in a cylindrical aluminum cup, which is fixed to the lift mounting platform. The cup with the motor can be moved vertically to mate with a four-stage pulley of the required diameter (16). Using a rubber belt, the electric motor rotates the micrometer drum. A metal disc with slots (14) is fixed on the movable plunger part of the micrometer. When the micrometer drum and the slitted disc rotate together, the optocoupler (15) reads the pulses generated when light passes through the slits in the disc. The number of pulses determines the angle of rotation of the micrometer and, hence, the amount of bending of the specimen. The rotation speed of the micrometer drum is set both by pulse width modulation of the motor power supply and by changing the position of the rubber belt on the four-stage pulley (16) mounted on the micrometer. In this way, it is possible to set the number of bending steps required, the magnitude of the bending, and also to set the operating algorithm for carrying out any cyclic and variable loading, for example, in experiments to study the fatigue of the specimen.

Since the elasticity and viscosity of plant samples depend on humidity and temperature, the sample holder was enclosed in a thermostatically controlled chamber (17). The chamber, which has no mechanical contact with the holder, is independently mounted on the mounting platform and can be raised and lowered synchronously with the sample by a screw lift (10). The chamber has the minimum volume (1500 cm^3^) required for the placement of the sample holder, temperature regulating elements, and temperature and humidity sensors. The micrometer, moving mechanisms, and scales remain outside the chamber. Four Peltier thermomodules TEC1-12707 (Moscow, Russia), located in the end wall of the chamber, were used to control the temperature in the chamber. The hot and cold surfaces of the thermomodules are in close thermal contact with heat exchangers, one of which is an air needle radiator (18), located inside the chamber, and the other is a tap water cooled radiator, located outside the chamber (19). The Arduino Uno R3 was used as the temperature controller, which controls the on/off switching of power through Peltier modules based on data from the BME 280 (Bosh, Gerlingen, Germany) temperature and humidity sensor (20). Temperature and humidity values are indicated on a small digital display (21). The temperature in the chamber can be set and maintained in the range of −5 to +50 °C. Petri dishes containing water or silica gel were placed in the chamber to achieve the required humidity. The humidity was also controlled using an Arduino Uno R3 and a BME280 sensor. The time of a specimen analysis was around 5 min. Water loss of a stem segment through the experiment was checked by sample weight and did not exceed 2% for the whole stem segment and 6% for the isolated xylem part.

Thus, the device for the inverse three-point bending test, realized in the above-described design, allows us to investigate the elastic modulus of various samples, including plant stems. The main advantage of using the inverted method is the high accuracy of bending value determination (up to 10 µm) and load value measurement (up to 0.001 N). This makes it possible to measure soft parts of plant stems with a low modulus of elasticity. If required, the instrument can be easily adapted for tensile tests. Small modifications to the V-shaped sample holder also allow a three-point test to be carried out on the different parts of the whole living plant. The Supplementary File S1 contains photos of the created device from various angles, as well as the programming code for the Arduino. Supplementary File S2 contains a list of the main components of the device with current prices and links to purchase them.

### 3.2. Test of the Device for the Inverse Three-Point Bending Test

#### 3.2.1. Adequacy of Mechanical Property Values Obtained with the Device

**Figure 3:**
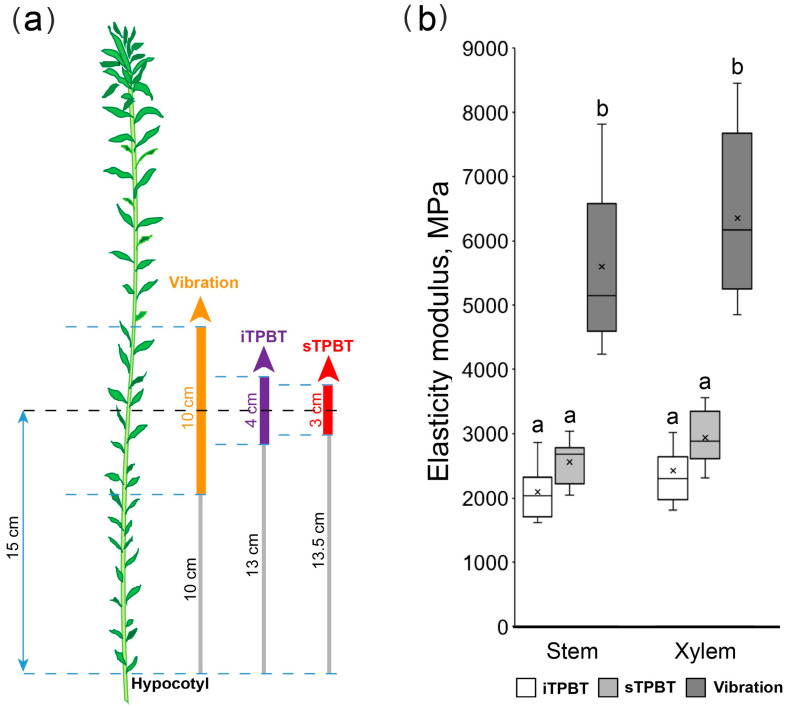
Comparison of three methods for investigating the macromechanical properties (iTPBT—inverse three-point bending test; sTPBT—straight three-point bending test; and vibration method). (**a**)—scheme of sample fixation. Color areas designate segments taken for mechanical properties study; gray areas are indents from the hypocotyls; (**b**)—elasticity moduli of investigated samples. The middle line is the median, the top box is the 75th percentile, the bottom box is the 25th percentile, the whiskers are the maximum and minimum values, and the black cross is an average value. Different letters above the boxes within the same part of the stem correspond to a significant difference according to one-way ANOVA followed by Tukey test at α = 0.01.

To test the proposed device, the values of elastic moduli in flax stem segments were determined by the inverse three-point bending test (iTPBT) and compared with the results of the straight three-point bending test (sTPBT) and the vibration method (Figure 3). Measurements were performed first on the defoliated stem segment and then on its xylem part, obtained after peeling off the phloem part. The plants (Bolshoy variety) were 48 days old after sowing and 40 cm high. The length of the segments necessary for different methods varied, and the samples were taken at various distances from the hypocotyl so that the middle of the samples coincided (Figure 3a).

Encouragingly, there were no differences in the values obtained by the inverse three-point bending test (Figure 3b, iTPBT) and the straight three-point bending test (Figure 3b, sTPBT); the latter was performed with a MultiDrive MCR 702e rheometer (Anton Paar, Austria) equipped with a load cell and linear actuator. The elasticity moduli were at the level of 2–3GPa and were similar for the stem segment as a whole and for its xylem part, being in the range described previously for similar samples [28,29] and lower than of mature flax stem where the modulus gets one order of magnitude higher [27].

Together with that, there were differences between the results obtained by the vibration method and both three-point bending tests (Figure 3b). Differences in mechanical properties using these approaches were also observed in the study of Arabidopsis inflorescence stems [10,26]. There are several possible explanations for this. Typically, bending tests allow direct calculation of the modulus, but they require an expensive machine equipped with a load cell and linear actuator. In contrast, vibration testing can be performed using relatively inexpensive tools, but the modulus is estimated indirectly based on the natural frequency of vibration [10]. In the vibration test, a prerequisite for estimating *k* is that the tested stems are straight and have a uniform mass distribution [10]. In the three-point bending test, the ovality of an inflorescence stem in the transverse plane can negatively affect modulus estimations [32]. In our case, the differences between the values can be explained, among other things, by the different experimental conditions. The three-point tests were conducted in chambers with humidity and temperature control, whereas the vibration test was conducted in air. The evaporation of moisture from the sample could have led to a change in the elasticity modulus of the investigated system [15,33]. However, the obtained values did not differ by orders of magnitude, and the trend of mechanical property distribution was maintained (Figure 3b).

#### 3.2.2. Sensitivity of the Device

**Figure 4:**
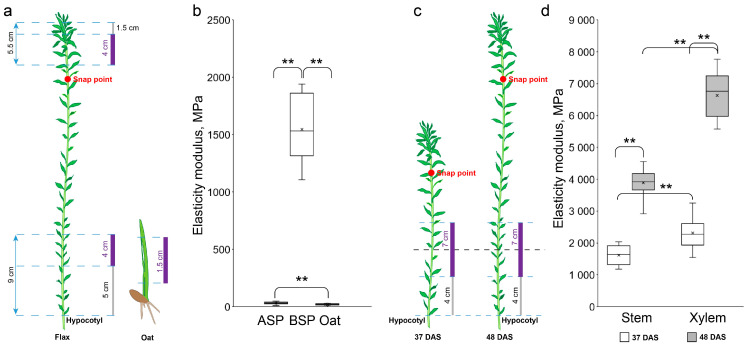
Comparison of oat coleoptile and flax stem segments with very different mechanical properties. (**a**)—scheme of sample fixation for different “skeleton” types. Purple areas are the segmentsused for the mechanical properties study;gray areas are indents from the apices/hypocotyl. (**b**), (**d**)—elasticity moduli of investigated samples; (**c**)—scheme of sample fixation for different plant ages. ASP—above the snap point, BSP—below the snap point, DAS—days after sowing. The middle line is the median, the top box is the 75th percentile, the bottom box is the 25th percentile, the whiskers are the maximum and minimum values, and the black cross is an average value. **—significant difference according to *t*-test at α = 0.01.

To verify the sensitivity of the device, samples with completely different mechanical properties were examined. Samples of flax stem of the variety Mogilevsky were taken above the snap point (1.5 cm from the apex) and below the snap point (5 cm from the hypocotyl) (Figure 4a). The upper section is characterized by the presence of predominantly cells with primary cell walls. The mechanical stability of this segment is maintained by a hydrostatic mechanism, which is based on the creation of turgor pressure in each cell and the resistance to it by the primary cell wall. In turn, the lower segment is characterized by the presence of fibers with powerfully developed secondary and tertiary cell walls. The deposition of thickened cell walls in specialized tissues qualitatively changes the parameters of the mechanical properties of tissues. Therefore, it is very difficult to evaluate mechanical properties by a single method due to the very large variation in values.

When evaluated with the constructed device, the region above the snap point possessing mostly primary cell walls had a low elasticity modulus (34.9 ± 10.7 MPa; Figure 4b, ASP). These values correlate with data obtained using other approaches for samples containing mainly cells with primary cell walls [22,34,35,36]. The segment below the snap point had an elastic modulus of 1546.7 ± 294.0 MPa (Figure 4b, BSP). The sharply different values mean that the distributed (hydrostatic) type of “skeleton” changes to an accentuated one, with a predominant role of the thickened cell walls developed in some specialized tissues.

Investigations were carried out in the range of the limit of the ratio of the sample length to its diameter [15,37]. The flax stem fragments were 4 cm long, and the ratio of the length to the diameter was about 20. The coleoptile fragments were 1.5 cm long, and the length-to-diameter ratio was 10. The latter values are the lower limit for working without corrections [37]. The Young’s modulus obtained for coleoptiles was 20.4 ± 6.3 MPa (Figure 4b, Oat). The coleoptile fragments, as well as the flax stem fragment above the snap point, were characterized by the presence of thin primary cell walls. The results were, therefore, within reasonable limits (Figure 4b, ASP and Oat). Moduli of elasticity obtained for different fragments demonstrate the sensitivity of the proposed device to values differing by orders of magnitude.

Another experiment demonstrating the sensitivity of the device was to investigate the mechanical properties of the stem at different stages of flax plant development (Figure 4c). Using the vibration method, it has been established that during the development of the flax stem in the long-fiber cultivar Mogilevsky, the mechanical properties of the segment below the snap point increased due to an increase in xylem area [29]. A similar situation was observed when evaluating mechanical properties of the flax stem in oilseed variety Bethune using the inverse three-point bending test (Figure 4b). Besides, the values 24.2 ±2.5 GPa were obtained for the segments of the whole stems from mature plants of Mogilevsky cultivar, which are very close to the obtained by sTPBT data of Requile with colleagues for mature flax stem from another long-fiber cultivar [27]. Thus, altogether, the device demonstrated good sensitivity and reliability for elasticity moduli determination in the range from 20 MPa to 25 GPa.

## 4. Conclusions

We introduce the novel device for mechanical property determination, which is particularly suitable for the axial organs of herbaceous plants. The design of the instrument is based on the three-point bending test methodology and uses its inverted variant: instead of applying aload and measuring the amount of deflection, the deflection is dictated by a precise micrometer and the load required to achieve such deflection is measured by portable analytical scales. The proposed version has both the advantages of three-point bending tests in general and some specific ones. Clamping of the specimen is not required, thus avoiding the associated problems. The constructed device demonstrates values matching those determined by the expensive analogs used for straight three-point bending tests. At the same time, the cost of the developed device is approximately one order of magnitude cheaper than that of commercial devices. The main advantage of the inverted method is high accuracy of bending value determination (up to 10 µm) and load value measurement (up to 0.001 N). The sensitivity of the device allows investigating short (from 1.5 cm) samples with elastic moduli in the range from tens of MPa to tens of GPa. The ability to maintain constant humidity and temperature allows us to minimize changes in the condition of samples. The measurements take several minutes for each specimen, without significant loss of water from the sample. The proposed design also helps to easily determine the moment of the first contact between the specimen and the sensor, which is convenient to start the measurement. The data from the scales are collected in digital form, which helps to easily convert them into stiffness values immediately after the experiment. Automation systems and video-recording allow to simplify the experiments and to eliminate errors and inaccuracies. The obvious disadvantage of the method is the current unavailability of a commercial version of the device. Classical difficulties with the shape and size of plant samples can be solved by variations in the chamber and holder dimensions. Minor modifications to the device may allow its future use to assess the relaxation behavior of the sample, making it possible to assess different components with different mechanical properties, such as the major tissues present in plant samples. Minor modifications to the specimen holder and chamber may also allow for a three-point bending test on the different parts of a whole living plant.

## Data Availability

Data supporting this study are included within the article.

## Supplementary Materials

The following supporting information can be downloaded at: https://www.mdpi.com/article/10.3390/mps8020032/s1, Supplementary S1: photos of the device, programme code and checklist for authors with recommendations; Supplementary S2: list of the basic components of the device.
