# Supplementary material for: Using the Inverse Three-Point Bending Test to Determine Mechanical Properties of Plant Stems
Source: Methods Protoc. 2025 Mar 18;8(2):32. doi: 10.3390/mps8020032 (PMC11932232; doi:10.3390/mps8020032)
Supplement: Supplementary file 1 [file mps-08-00032-s001.zip › Supplementary S1.pdf]

Photos of the reverse three-point bending test machine made from different angles. The numbers in the images match the numbers in Figure 2 in the main text. This file also includes a sketch for Arduino and checklist for authors with recommendations when manufacturing device.

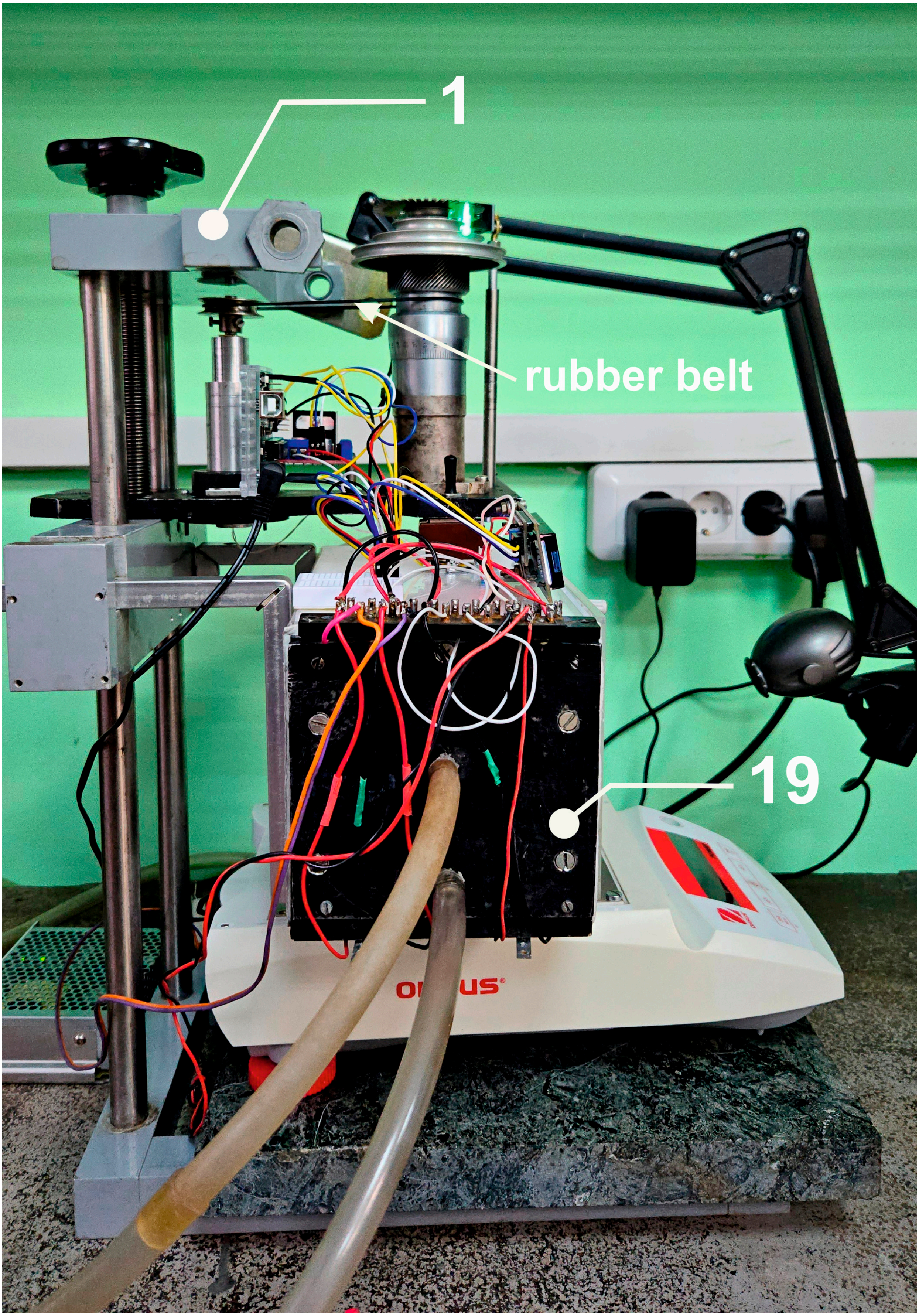

View from the left

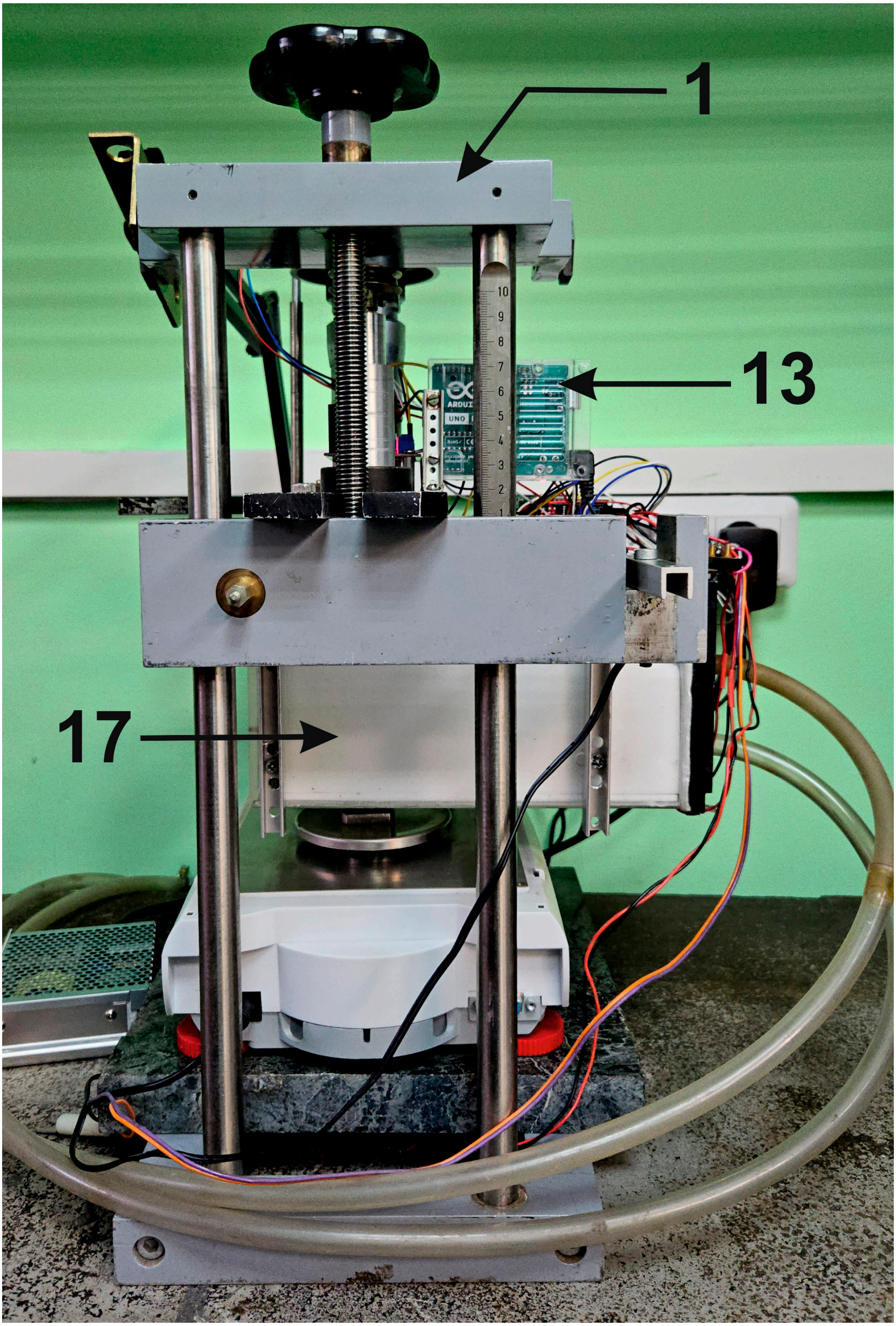

View from the back

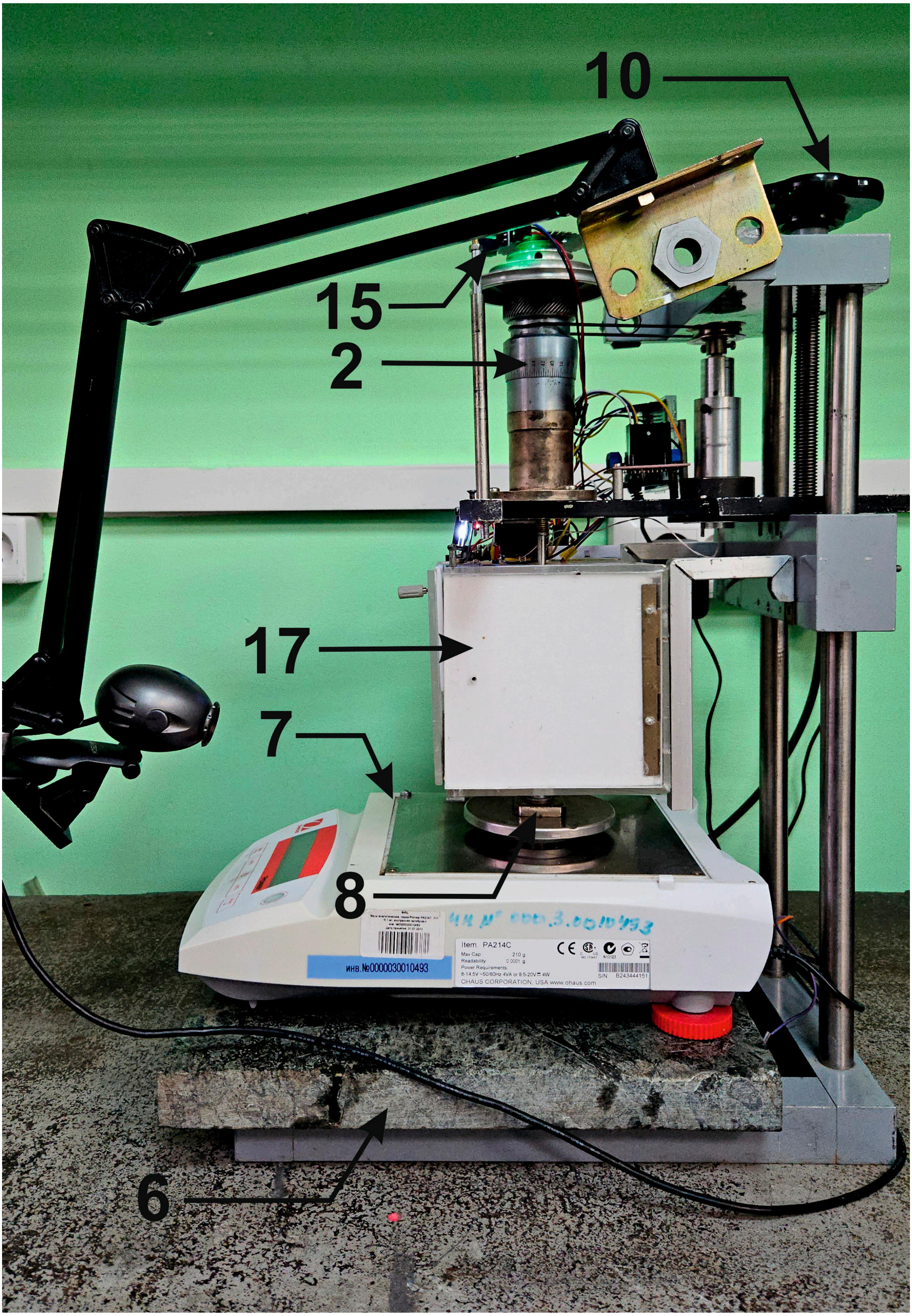

View from the right

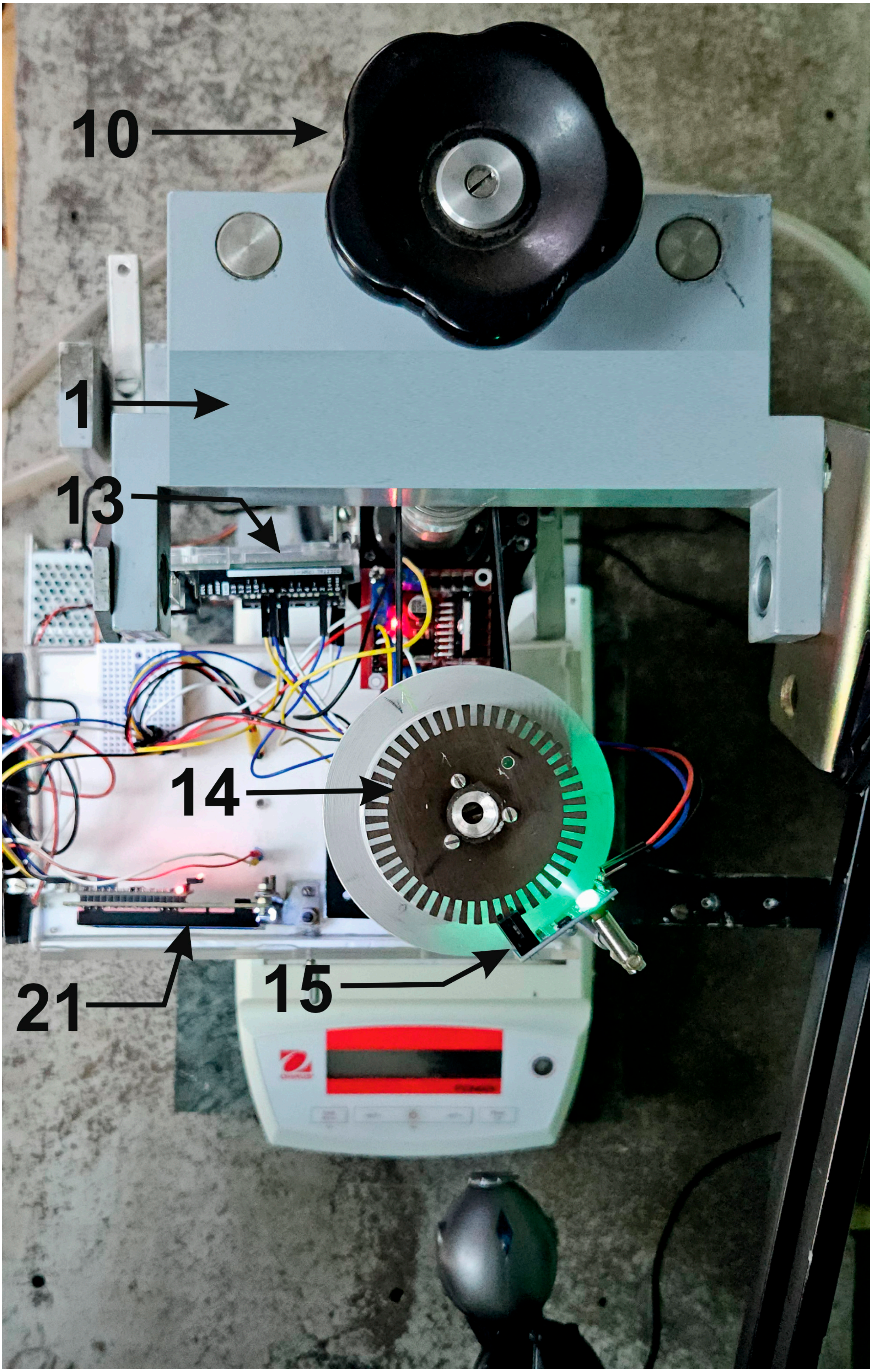

View from the top

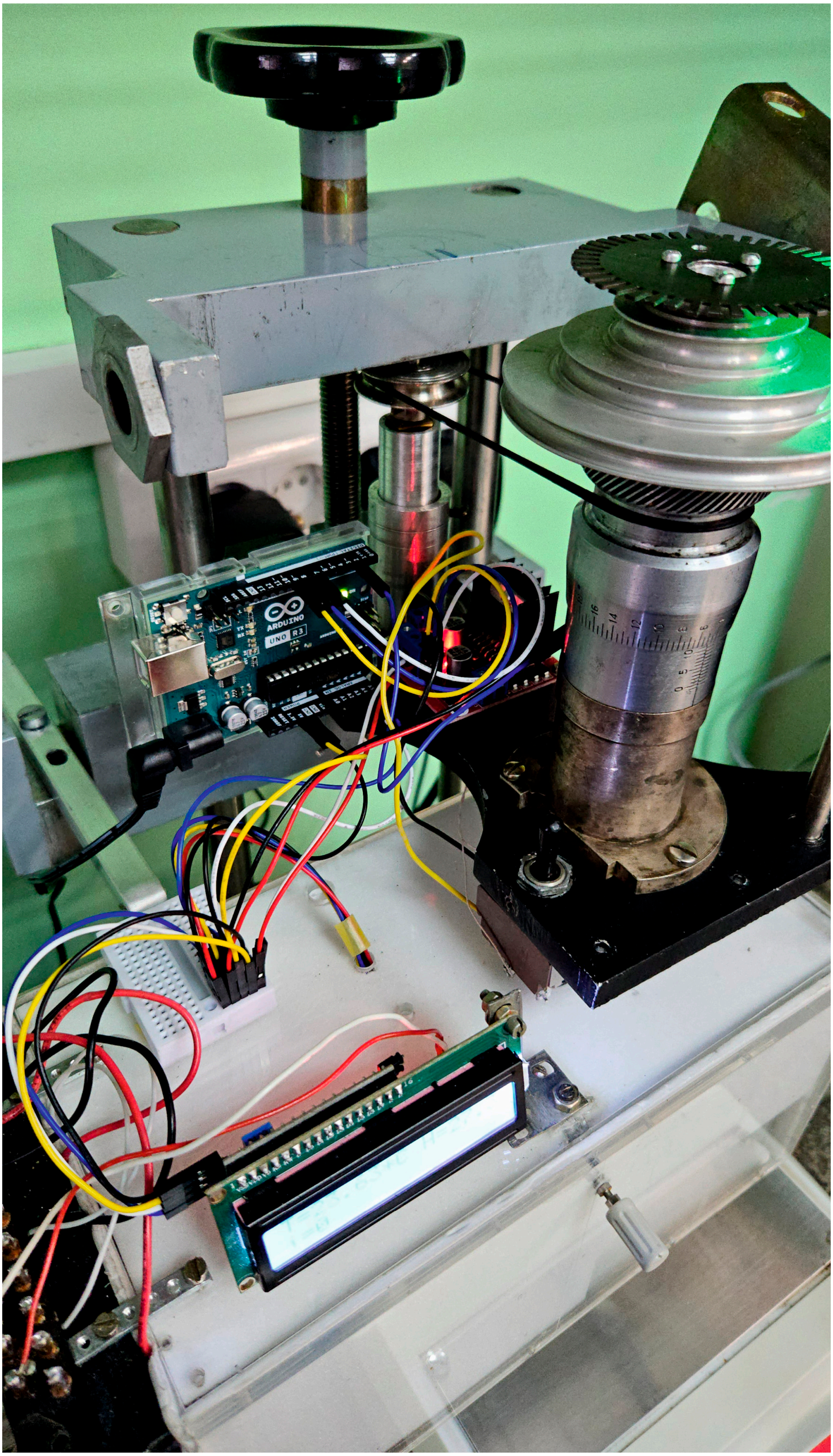
ARDUINO
UNO R3

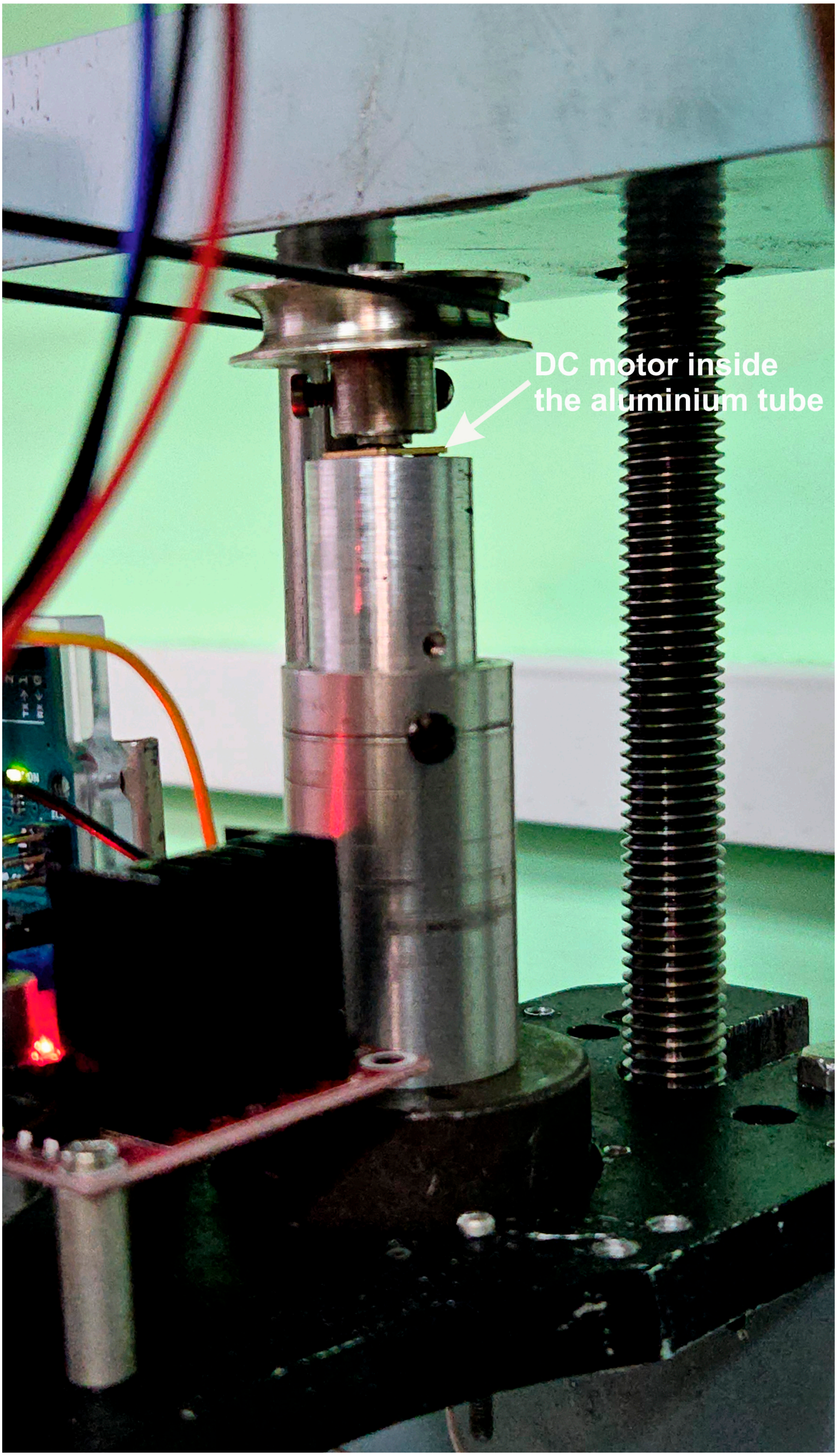
DC motor inside
the aluminium tube

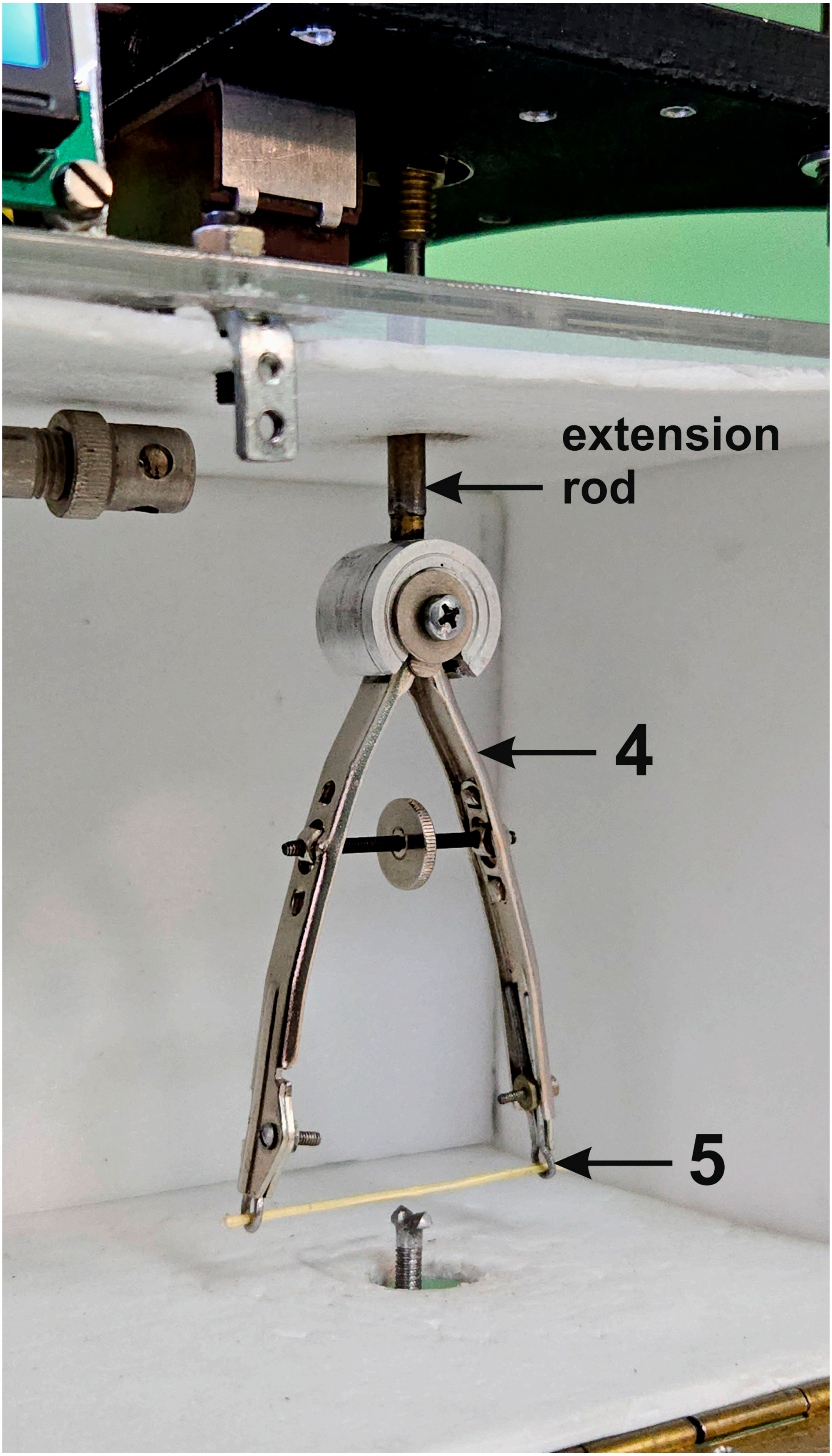
extension rod
4
5

To ensure the accuracy and stability of measurements, it is desirable to fulfil the following recommendations when manufacturing the device:

1) exclude the possibility of uncontrolled displacement of the platform on which the micrometer is installed (is provided by the screw lift);

2) achieve a tight contact between the movable micrometer plunger (3) and the extension rod on which the V-shaped sample holder (4) is fixed (by springing the rod to the movable plunger);

3) achieve a firm fit of the V-shaped holder on the rod;

4) check that the scales do not sag when loaded. It is better to use a load cell scale for this purpose;

5) exclude the possibility of vibrations of the device using a heavy base;

6) other details of the device allow different solutions depending on the capabilities of the experimenter.

Programme code for Arduino (sketch):

```
#include <SoftwareSerial.h>
#include <LiquidCrystal_I2C.h>
#include <Adafruit_BME280.h>
LiquidCrystal_I2C lcd (0X27,16,2);
Adafruit_BME280 BME280; //initialising of temperature and humidity sensor
intenA = 9;
int in1 = 8;
int in2 = 7;
const byte interruptPin = 2;
int counter = 0; //optical encoder pulse counter
constintstep_number=10; // number of sample loading steps
int k=0;
inttotalCounter=0;
intPauseTime=10000; //pause between load feeds

void detect() { // interrupt handling procedure
   counter++;
}
void setup() {
Serial.begin(9600);
lcd.init();
lcd.backlight();
lcd.print("Starting");
pinMode(enA, OUTPUT);
pinMode(in1, OUTPUT);
pinMode(in2, OUTPUT);
digitalWrite(in1, LOW);
digitalWrite(in2, LOW);
pinMode(interruptPin, INPUT_PULLUP);
attachInterrupt(digitalPinToInterrupt(interruptPin), detect, RISING);
  k=0;
  counter=0;
lcd.clear();
lcd.setCursor(0, 0);
lcd.print("T=");
lcd.print(String(BME280.readTemperature())); // output to LCD the temperature value in the chamber
lcd.print("*C");
lcd.setCursor(0, 1);
lcd.print("H=");
lcd.print(String(BME280.readHumidity())); //output to LCD the humidity value in the chamber
lcd.print("%");
lcd.setCursor(1, 0);
lcd.print("k=");
lcd.print(String(k)); //output to LCD the current step of sample loading
}

void loop() {
  if (k<step_number){
analogWrite(enA, 255);
   counter=0;
```

```
   while (counter <50)// setting value is the number of pulses (interruptions), which corresponds to the
necessary rotation of the disc with slots and, accordingly, of the micrometer, to achieve the specified value
of the sample bending at one loading step
     {
digitalWrite(in2, HIGH);
digitalWrite(in1, LOW);
     }
digitalWrite(in2, LOW);
digitalWrite(in1, LOW);
    k+=1;
totalCounter+=counter;
lcd.clear();
lcd.setCursor(0, 0);
lcd.print("T=");
lcd.print(String(BME280.readTemperature())); // output to LCD the temperature value in the chamber
lcd.print("*C");
lcd.setCursor(0, 1);
lcd.print("H=");
lcd.print(String(BME280.readHumidity())); //output to LCD the humidity value in the chamber
lcd.print("%");
lcd.setCursor(1, 0);
lcd.print("k=");
lcd.print(String(k)); //output to LCD the current step of sample loading
    delay (PauseTime);
    if (k=step_number) {
        counter=0;
        while (counter<totalCounter)  //sample unloading by reversing the motor
        {
digitalWrite(in2, LOW);
digitalWrite(in1, HIGH);
        }
totalCounter=0;
digitalWrite(in2, LOW);
digitalWrite(in1, LOW);
        }
  }
}
```
