# Supplementary material for: Using the Inverse Three-Point Bending Test to Determine Mechanical Properties of Plant Stems
Source: Methods Protoc. 2025 Mar 18;8(2):32. doi: 10.3390/mps8020032 (PMC11932232; doi:10.3390/mps8020032)
Supplement: Supplementary file 1 [file mps-08-00032-s001.zip › Supplementary S2.pdf]

The basic components needed for the inverse three-point bending test machine, with the actual prices and links.

№	Number of component in Figure 2	Name	Price, USD	Purchase link
1	1	Cremalier (lift platform)	238	https://aliexpress.ru/item/1005007494074619.html?sku_id=12000041015137743&spm=a2g2w.productlist.search_results.10.566050b3Xu9n2D
2	2	Mechanical micrometer	9.47	https://aliexpress.ru/item/1005004856953604.html?spm=a2g2w.detail.rcmdprod.4.e8426398XJQsQo&mixer_rcmd_bucket_id=aerabtestalgoRecommendAbV2_testRankingNewFeaturesItemContext&pdp_trigger_item_id=0_32864886329&ru_algo_pv_id=e049ab-61b690-edce44-6d7b4d-1741593600&scenario=aerSimilarItemPdpRcmd&sku_id=12000030769893710&traffic_source=recommendation&type_rcmd=core
3	4	H2031 Spring compasses divider multifunctional (for V-shaped sample holder)	2	https://aliexpress.ru/item/1005005954421151.html?sku_id=12000035010870924&spm=a2g2w.productlist.search_results.4.73f2571eBnsD53
4	7	Analytical scales	2500	https://us.ohaus.com/en-us
5	14	Disc with slots (for optical sensor)	0.23	https://kulibin.su/catalog/radiodetali/arduino-moduli/opticheskie-datchiki/disk-dlya-kontsevogo-opticheskogo-datchika.html
6	15	1PCS Beam Photoelectric Sensor With Infrared Sensor Module (optocoupler)	0.24	https://aliexpress.ru/item/32975445830.html?sku_id=66737609833&spm=a2g2w.productlist.search_results.6.72e83cc7cIIYEq
7	13	Arduino UNO REV3	25.16	https://aliexpress.ru/item/1005007219507247.html?sku_id=12000039850054523&spm=a2g2w.productlist.search_results.1.72af68aeOCzuJL
8	21	LCD Module 1602 lcd i2c	1.74	https://aliexpress.ru/item/32287272967.html?sku_id=17616533880&spm=a2g2w.productlist.search_results.2.7fb06c52GPatRZ
9	Supplementary S1	5V12V Power Box RD-65A	30.58	https://aliexpress.ru/item/1005006822396787.html?sku_id=12000039048689857&spm=a2g2w.productlist.search_results.2.9c93152ft2Ebuo
10	12	GA12-N20 DC 6V 30RPM (DC motor with a gearbox)	1.75	https://aliexpress.ru/item/1005005393396406.html?sku_id=12000043386932389&spm=a2g2w.productlist.search_results.11.5cf5db18Gnb11k
11	Supplementary S1	L298N Motor Driver Controller Board L298	1.34	https://aliexpress.ru/item/1005008123620825.html?sku_id=12000044096012738&spm=a2g2w.productlist.search_results.0.25d06518BQGZBz
12	Supplementary S1	Belts	2.82	https://aliexpress.ru/item/1005004879913041.html?sku_id=12000030868845854&spm=a2g2w.productlist.search_results.3.c94c61a5DMkO55
13	20	BME280 5V 3.3V Digital Sensor Temperature Humidity	2.18	https://aliexpress.ru/item/1005001827151118.html?sku_id=12000017775153915&spm=a2g2w.productlist.search_results.0.44025bafLvY9fC

		Barometric Pressure Sensor Module I2C SPI 1.8-5V		
14	18	100x100x18mm radiator	8.68	https://aliexpress.ru/item/32239661224.html?sku_id=50601615994&spm=a2g2w.productlist.search_results.2.f34966f3qrMkZt
15	Not shown in Figure 2	TEC112706Thermoelectric Peltier Elemente Module 40MM 12V	1.90	https://aliexpress.ru/item/32822435740.html?sku_id=12000024124026755&spm=a2g2w.productlist.search_results.3.673a41e3h85n7G

Total price: $2826 U.S.

Further material costs are to be anticipated for the manufacture of the thermostatically controlled chamber (made of polymethylmethacrylate), sample prism and sample ring holders, as well as for the purchase of connecting wires and hoses for the cooling system, stopwatch, web-camera. Please be advised that the additional cost may amount to $100.

The price of a universal tree-point bending test machines ranges from $12 900 to $150 000. The 3 main factors that affect the cost are force test space and controller with force capacity having the most impact.

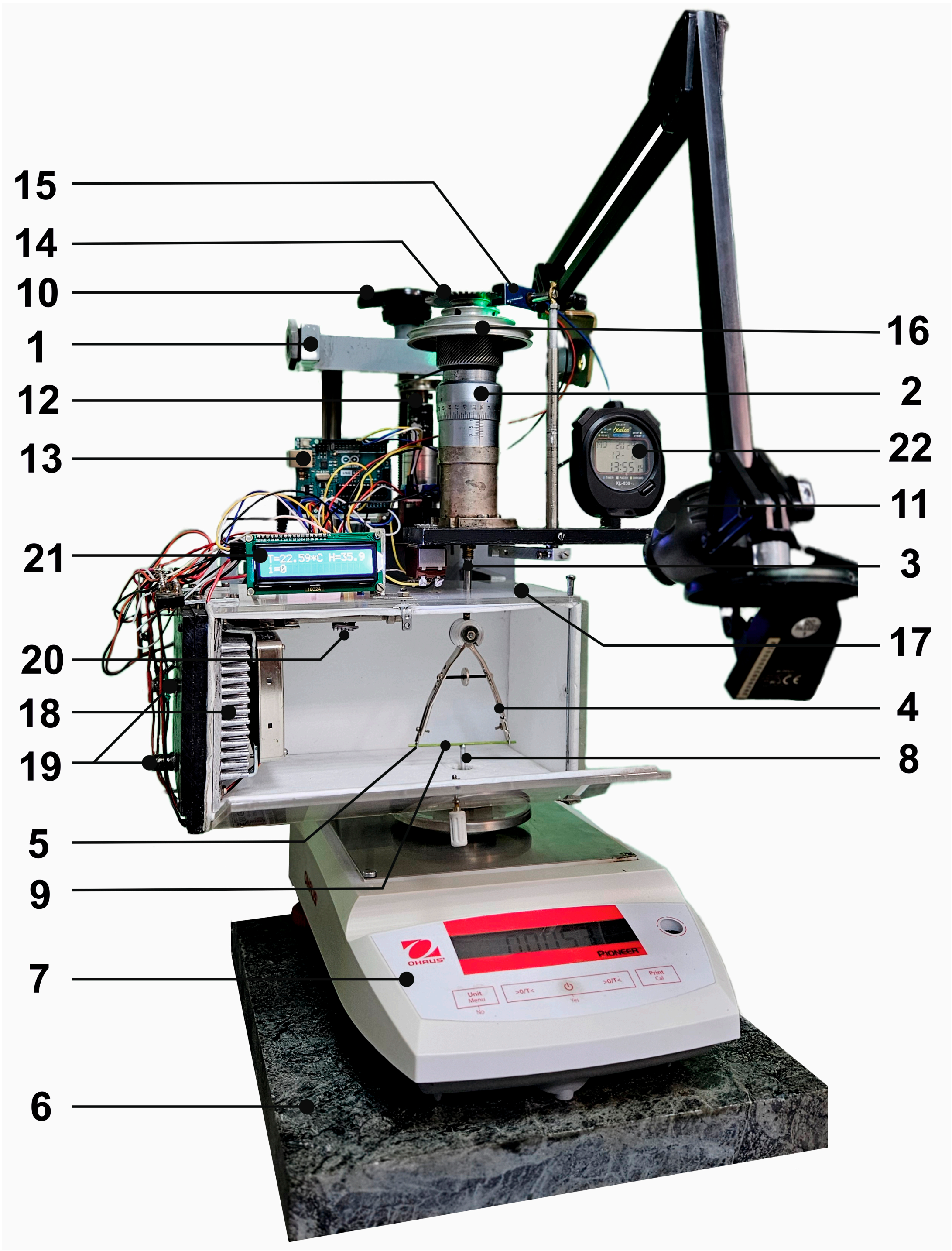

Figure 2. The design of the device for inverse tree-point bending test: 1 – cremalier; 2 – mechanical micrometer; 3 – movable plunger; 4 – V-shaped sample holder; 5 – stem segment holding rings; 6 – vibration-absorbing ceramic granite plate; 7 – analytical scales; 8 – support prism; 9 – stem segment; 10 – screw lift; 11 – video camera; 12 – DC motor with a gearbox; 13 – ArduinoUno R3 hardware and software platform; 14 – metal disc with slots; 15 – optocoupler; 16 – four-stage pulley; 17 – thermostatically controlled chamber; 18 – needle radiator; 19 – water cooled radiator; 20 – temperature and humidity sensor; 21 – digital display; 22 – stopwatch.
